# High-intensity intermittent training promotes adipose tissue browning via the IL-27/p38 MAPK–PGC-1α signaling pathway in diet-induced obese rats

**DOI:** 10.3389/fphys.2026.1745363

**Published:** 2026-02-25

**Authors:** Chunlong Wang, Yulong Hu, Junfei Chen, Yihan Wu

**Affiliations:** 1 College of Physical Education, Yangzhou University, Yangzhou, China; 2 Jiangsu Institute of Sports Science, Nanjing, China

**Keywords:** adipose tissue browning, high-intensity intermittent training, il-27, obesity, UCP1

## Abstract

**Objectives:**

This study investigated the effects of high-intensity intermittent training (HIIT) *versus* moderate-intensity aerobic training (MAIT) on IL-27 signalling and adipose tissue browning in obese rats.

**Methods:**

Forty male Sprague–Dawley rats were randomly divided into two groups: standard diet (C, n = 10) and high-fat diet (HFD, n = 30). After 8 weeks of HFD feeding, 24 obese rats were further randomised into three subgroups: HFD (H, n = 8), HFD + moderate-intensity training (HMT, n = 8), and HFD + HIIT (HHT, n = 8). The HMT and HHT groups underwent 8 week training interventions (six sessions/week). The HMT protocol included a 10 min warm-up (treadmill speed: 10 m/min), a 40 min moderate-intensity aerobic phase (60%–70% of maximum speed), and a 10 min recovery (10 m/min). The HHT protocol featured 10 min warm-up and recovery phases (10 m/min), with 40 min of alternating treadmill training: 3 min at 50% maximum speed followed by 3 min at 90% maximum speed.

**Results:**

No significant differences in body weight were observed between the HHT and HMT groups. HHT rats displayed significantly lower plasma triglyceride levels than H and HMT rats. Compared with HMT, HHT reduced adipose mass and adipocyte size and increased mitochondrial succinate dehydrogenase and cytochrome c oxidase (COX) activities in adipose tissue. However, HHT rats displayed lower COX activity in visceral white adipose tissue than HMT rats. Training upregulated browning-related genes and uncoupling protein 1 (UCP1) in adipose tissue, with stronger effects in HHT than in HMT. Plasma and adipose tissue IL-27 levels, as well as p38 MAPK–PGC-1α signalling pathway activation, were significantly elevated in both training groups, with greater increases in HHT.

**Conclusion:**

HIIT promotes adipose tissue browning by activating the IL-27 signalling pathway and ameliorates obesity-associated metabolic disorders more effectively than MAIT, supporting its potential as a therapeutic strategy for obesity.

## 1 Introduction

Obesity is a chronic, progressive disease characterised by low-grade inflammation, marked by increased expression of pro-inflammatory cytokines and reduced expression of anti-inflammatory cytokines (Yi et al., 2020; Chang et al., 2021). It is strongly associated with hypertension, hyperlipidaemia, type 2 diabetes, colorectal tumours, other cancer types, and neurodegenerative diseases (Alford et al., 2018; Leggio et al., 2017; Kim et al., 2019; Chen et al., 2019; Liu et al., 2019). Consequently, obesity has been recognised as a disease state by the American Medical Association and other organisations and thus has become a global health concern. Although pharmacological interventions are available, their adverse side effects limit application, making exercise a more favourable strategy.

Adipose tissue is categorised into white adipose tissue (WAT) and brown adipose tissue (BAT) based on adipocyte size, lipid droplet content, mitochondrial abundance, and uncoupling protein 1 (UCP1) levels. BAT is an energy-expending tissue characterised by smaller lipid droplets and abundant mitochondria. The transformation of WAT into BAT, termed WAT browning (Yu et al., 2015), is regulated by multiple molecular mechanisms and can be triggered by stimuli such as exercise, cold exposure, fasting, and bariatric surgery (Wang et al., 2021; Yiliang et al., 2024). IL-27 directly acts on adipocytes through its receptor IL-27Rα, activating p38 MAPK and driving the expression of PGC-1α and UCP1. In Adipoq-Cre or Ucp1-CreERT2-mediated conditional knockout mice of IL-27Rα, the metabolic protective effects of IL-27 are significantly weakened or lost. Mice lacking IL-27Rα or EBI-3 are more prone to obesity, insulin resistance, and fatty liver disease (Wang et al., 2021).

Exercise exerts dual effects on cytokine secretion: Acutely, it initiates local inflammatory responses to facilitate repair; chronically, it reduces chronic inflammation by stimulating skeletal muscle to release myokines such as interleukin-6 (IL-6), IL-10, and IL-1 receptor antagonist (IL-1RA). However, the molecular mechanisms underlying exercise intensity-dependent adipose browning remain incompletely understood. Notably, IL-27 secretion is elevated in inflamed adipocytes (Nam et al., 2016), and IL-27 can activate the p38 mitogen-activated protein kinase (p38 MAPK)-peroxisome proliferator-activated receptor gamma coactivator 1-alpha (PGC-1α) signalling axis, thereby upregulating UCP1 expression (Wang m.fl. 2021) and enhancing adipose thermogenesis. This study investigated the effects of high-intensity intermittent training (HIIT) and moderate-intensity aerobic training (MAIT) on systemic inflammation, IL-27 expression, and adipose browning in rats with high-fat diet (HFD)-induced obesity.

## 2 Materials and methods

### 2.1 Ethics statements

This study was reviewed and approved by the Institutional Animal Care and Use Committee of Yangzhou University (Protocol No. 202407037).

### 2.2 Animals and diets

Forty male Sprague–Dawley rats, aged 6–8 weeks, were selected as experimental subjects. The animals were obtained from the Jiangsu Experimental Animal Centre of Nanjing Medical University (production licence number: SYXK [Jiangsu] 2023–0081). They were housed in groups of four per cage under controlled environmental conditions: room temperature 20 °C–26 °C, relative humidity 40%–70%, a 12 h light-dark cycle, and *ad libitum* access to food and water. After a 1-week acclimatisation period on standard chow (Synergy: 1,010,088), an 8 week obesity induction phase was initiated. Rats (aged 7–9 weeks) were randomly divided into two groups: control (C, n = 10) and high-fat diet (HFD, n = 30). The control group continued to receive standard chow, whereas the HFD group was fed a high-fat diet (Synergy: XTHF45-1) containing 47% of calories from fat, 20% from protein, and 33% from carbohydrates. Following the 8 week obesity induction, rats in the HFD group with a Lee’s index 20% higher than the mean value of the control group and a Lee’s index ≥310 were classified as obese (Lee, 1929). Twenty-four obese rats (aged 15–17 weeks) meeting these criteria were randomly assigned into three subgroups: high-fat diet (H, n = 8), high-fat diet + aerobic training (HMT, n = 8), and high-fat diet + high-intensity intermittent training (HHT, n = 8). All subgroups continued their respective diets (standard or high-fat) until the conclusion of the experiment. Clean the animal room and cages daily, and replace the drinking water. Record and calculate the difference between the initial food supply and the remaining amount the next day for each cage of rats to obtain the food intake. Replace the bedding once every 2 days.

### 2.3 Experimental protocol

Following a 1-week adaptive training period, rats in the HMT and HHT groups underwent an 8 week training intervention (six sessions/week) beginning in Week 10, whereas no training intervention was applied to the C and H groups. Body weight and food intake were recorded daily throughout the experiment. Twenty-four hours after the final training session. Following induction with 3%–4% isoflurane anesthesia via inhalation (maintained at 2%–2.5%), the experimental rat was placed in a supine position and secured. Blood was collected via femoral artery puncture. Subsequently, the chest was rapidly opened to expose the heart. The pericardium was incised using scissors, and a perfusion cannula was inserted into the left ventricle. Perfusion with ice-cold PBS was performed rapidly until the liver blanched and the effluent became clear. After perfusion, the absence of spontaneous respiration and reflex activity confirmed that the animal was non-responsive. Tissue samples were then harvested. A portion of the tissue was reserved for detection of target gene/protein expression, while another portion was prepared for histological staining.

### 2.4 Maximum running speed test

At the start of the training intervention, an incremental-load treadmill test was performed to determine the rats’ maximal running speed. The treadmill incline was set to 0°, and the initial speed was 10 m/min, which was increased by 2 m/min at each stage until the rats reached exhaustion and could not continue running despite re-peated encouragements. The speed of the penultimate stage was recorded as the maximal running speed.

### 2.5 Exercise training

For the HMT group, each session comprised a 10 min warm-up phase (treadmill speed: 10 m/min), a 40 min moderate-intensity aerobic phase (60%–70% of maximal running speed), and a 10 min recovery phase (treadmill speed: 10 m/min).

For the HHT group, each session comprised a 10 min warm-up phase and a 10 min recovery phase (both at 10 m/min) at the beginning and end, respectively. The intermediate 40 min interval comprised alternating treadmill exercise: 3 min at 50% of maximal running speed followed by 3 min at 90%, repeated throughout the 40 min phase (Høydal et al., 2007).

### 2.6 Enzyme-linked immunosorbent assay

Assay kits for triglyceride (TG), total cholesterol (T-CHO), free fatty acid (FFA), cytochrome c oxidase (COX), and succinate dehydrogenase (SDH) activities were sourced from Nanjing Jiancheng Bioengineering Institute. IL-27 enzyme-linked im-munosorbent assay kits were obtained from Suzhou Jianglai Biotechnology Co., Ltd.

### 2.7 Hematoxylin and eosin (H&E) staining

The tissue samples were fixed, embedded in paraffin, and sectioned. After routine dewaxing and hydration, they were stained with hematoxylin for 6 min, differentiated with acid alcohol (1% HCl in 70% ethanol) for 3 s, blued in running water, counterstained with eosin for 2 min, and finally dehydrated, cleared, and mounted with neutral gum.

### 2.8 Quantitative real-time PCR analysis

Total RNA was extracted from iWAT and PRAT using TRIzol (Ambion, Texas, United States). RNA concentration was measured using an ultraviolet spectrophotometer, and cDNA was synthesised using the ReverTra Ace™ qPCR RT Kit (TOYOBO Co., Ltd., Osaka, Japan). qPCR was performed using the FastStart Universal SYBR Green Master Mix (Rox) kit (Roche Diagnostics Corporation, Indiana, United States), and relative gene ex-pression was calculated using the 2^−ΔΔCT^ method. The primer sequences for the target genes are shown in Table 1.

**TABLE 1:** Primers for real-time PCR.

Gene name	Forward sequences (5′-3′)	Reverse sequences (5′-3′)
GAPDH	5′-TGCCACTCAGAAGACTGTGG-3′	5′-TTCAGCTCTGGGATGACCTT-3′
UCP1	5′-GGGCTGATTCCTTTTGGTCTCT-3′	5′-GGGTTGCACTTCGGAAGTTGT-3′
Cidea	5′-TGTTAAGGAGTCTGCTGCGGTTC-3′	5′-ATGGCTGCTCTTCTGTGTCACC-3′
Cited	5′-AAGCCAACCAGGAGAGGATGAG-3′	5′-GGGCACCAGCAGGAGAGAC-3′
Tbx1	5′-GGCAGGCAGACGAATGTTCCC-3′	5′-CAGCCACCAGCCAGGAGGAG-3′
IL-27	5′-AGCAGACCCCCTGAGCCT-3′	5′-GTGGTAGCGAGGAAGCAGAGT-3′
PGC-1α	5′-TATTCATTGTTCGATGTGTCGC-3′	5′-TGTCTGTAGTGGCTTGATTCAT-3′
P38-Mapk	5′- CACGAGAATGTGATTGGTCTGTTGG-3′	5′- CACTTCACGATGTTGTTCAGGTCTG-3′

### 2.9 Western blotting

Protein samples were separated by 10% sodium dodecyl sulphate polyacrylamide gel electrophoresis. The polyvinylidene difluoride membrane was activated, equili-brated, and placed in a rotary folder using the standard sandwich configuration for 10–15 min. The transferred proteins were detected by immunoblotting, which involved rinsing and blocking, followed by sequential incubation with a primary antibody against UCP1 (1:1,000, ABclonal) and an HRP-conjugated goat anti-rabbit IgG (H + L) secondary antibody (1:10,000, ABclonal). Signals were visualised by enhanced electro-chemiluminescence, and images were captured in greyscale.

### 2.10 Statistical analysis

Data were analysed using GraphPad Prism software (version 10.2) and are pre-sented as mean ± standard error of the mean (SEM). Comparisons between the C and H groups were performed using a two-tailed t-test and one-sample t-test. Differences among the H, HMT, and HHT groups were assessed by one-way analysis of variance followed by Tukey’s post hoc test. A p-value <0.05 was considered statistically significant.

## 3 Results

### 3.1 Changes in body weight and lipid metabolism

**FIGURE 1:**
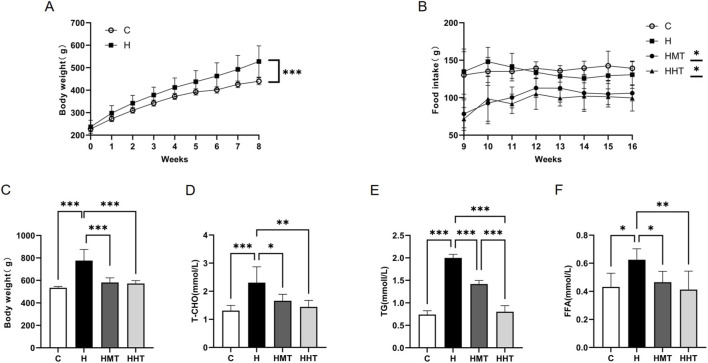
Body weight and lipid metabolism in rats at different stages. **(A)** Changes in body weight during the modelling phase. **(B)** Food intake and **(C)** body weight changes in rats during the intervention phase. **(D–F)** Effects of high-fat diet (HFD) and exercise training regimens on plasma T-CHO, TG, and FFA levels. Data are presented as mean ± SEM. Significant differences between groups are indicated as *p < 0.05, **p < 0.01, and ***p < 0.001.

After 8 weeks of HFD feeding, rats in the H group exhibited significantly higher body weight than those in the C group (Figure 1A). During the 8 week training intervention, a significant difference in food intake was observed between the exercise groups and the sedentary group. However, no significant difference was found between the HMT and HHT groups. (Figure 1B), whereas exercise training reduced overall body weight (Figure 1C). Following the 8 week intervention, rats in the HMT and HHT groups displayed lower body weight compared with the H group (Figure 1D). HFD feeding significantly increased plasma T-CHO, TG, and FFA, while exercise training enhanced lipid metabolic utilisation (Figures 1E,F). Notably, the HHT group showed greater reductions in plasma TG levels than the HMT group, an effect that may result from improved fat oxidation capacity following high-intensity intermittent training.

### 3.2 Adipose tissue weight and adipocyte size

**FIGURE 2:**
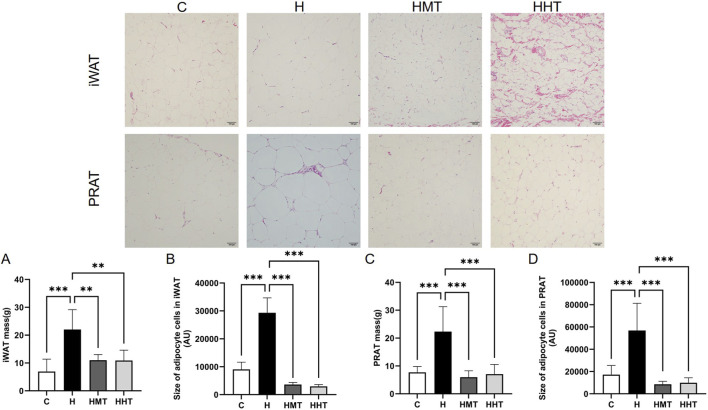
Morphology and weight of adipose tissue across experimental groups. Effects of high-fat diet (HFD) and different exercise training regimens on **(A)** inguinal adipose tissue weight, **(B)** inguinal adipocyte size, **(C)** perirenal adipose tissue weight, and **(D)** perirenal adipocyte size. Data are presented as mean ± SEM. Significant differences between groups are indicated as *p < 0.05, **p < 0.01, and ***p < 0.001.

After dissection, iWAT and PRAT were isolated and weighed. Results showed that obesity led to significant adipose accumulation, manifesting as marked increases in iWAT (Figure 2A) and PRAT (Figure 2C) weights, which were attenuated by exercise training. Similarly, adipocyte size in iWAT (Figure 2B) and PRAT (Figure 2D) increased in obese rats and decreased following exercise. No significant differences were observed between HIIT and MAIT in reducing adipose tissue weight or cell size, consistent with previous findings (Khalafi et al., 2020).

### 3.3 Mitochondrial respiratory enzyme activity

**FIGURE 3:**
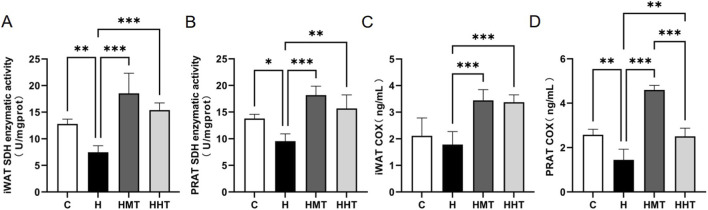
Mitochondrial activity in adipose tissue of rats across experimental groups. Effects of high-fat diet (HFD) and different training regimens on **(A)** succinate dehydrogenase (SDH) activity in inguinal adipose tissue, **(B)** SDH activity in perirenal adipose tissue, **(C)** cytochrome c oxidase (COX) content in inguinal adipose tissue, and **(D)** COX content in perirenal adipose tissue in rats. Data are presented as mean ± SEM. Significant differences between groups are indicated as *p < 0.05, **p < 0.01, and ***p < 0.001.

Analysis of cellular respiratory enzyme activity levels allows assessment of mitochondrial functional status. Complex II (or succinate dehydrogenase, SDH), encoded by nuclear DNA, is typically unaffected by mitochondrial DNA damage; increased SDH activity may indicate mitochondrial biogenesis (Edgar et al., 2009; Ross et al., 2010). Complex IV (or cytochrome c oxidase, COX) is essential for mitochondrial respiration (Ross, 2011). In this study, enzymatic activity assays of iWAT and PRAT revealed that obesity suppressed SDH activity in iWAT (Figure 3A), an effect reversed by training. Training also enhanced COX activity in iWAT (Figure 3C). SDH activity in PRAT (Figure 3B) followed the trend observed in iWAT. Obesity suppressed COX activity in PRAT (Figure 3D) but was restored with training. Collectively, training improved adipose mitochondrial function by enhancing SDH and COX activity, with greater improvements in iWAT than in PRAT. MAIT had stronger effects than HIIT in improving SDH and COX activity in iWAT and PRAT, potentially because MAIT primarily relies on fat oxidation as its main energy substrate.

### 3.4 Adipose tissue browning

**FIGURE 4:**
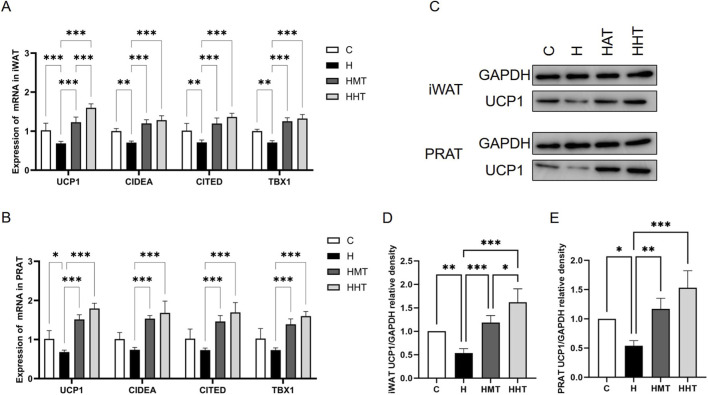
Adipose tissue browning in rats across experimental groups. Effects of obesity and exercise training on **(A)** mRNA expression of Ucp1, Cidea, Cited, and Tbx1in inguinal adipocytes and **(B)** perirenal adipocytes. **(C–E)** UCP1 protein expression in inguinal and perirenal adipocytes. Data are presented as mean ± SEM. Significant differences between groups are indicated as *p < 0.05, **p < 0.01, and ***p < 0.001.

UCP1 is a marker of adipocyte thermogenic capacity, and its expression is used to distinguish BAT from WAT (Sidossis and Kajimura, 2015). Results showed that obesity reduced UCP1 expression in iWAT and PRAT, indicating that obesity impairs adipose thermogenic capacity, whereas training restored UCP1 expression (Figures 4A,B). Training also enhanced adipose thermogenic capacity, with HMT exhibiting superior effects. The elevated expression of Cidea, Cited, and Tbx1, genes enriched in brown or beige adipose tissue, suggests that training promotes the conversion of WAT to beige or BAT.

At the protein level, UCP1 abundance in iWAT and PRAT decreased with obesity but increased after exercise training (Figures 4C–E). HHT was more effective than HMT in elevating UCP1 protein abundance in iWAT.

### 3.5 Levels of IL-27 and related regulatory pathways in adipose tissue

**FIGURE 5:**
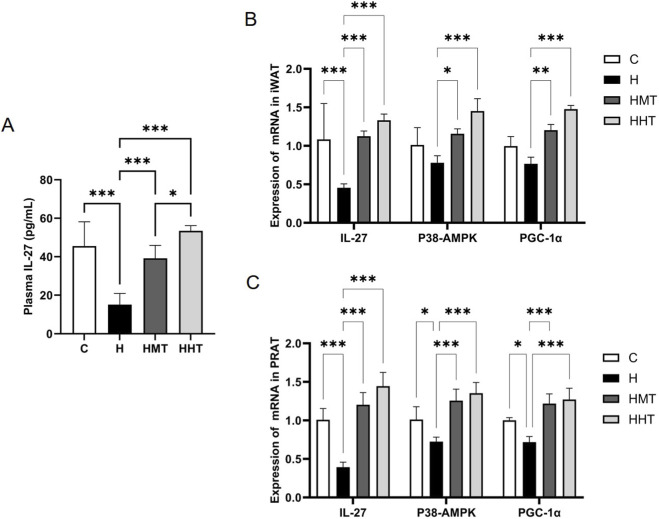
Plasma IL-27 levels and IL-27 signalling pathway expression in adipose tissue across experimental groups. Effects of obesity and exercise training on **(A)** plasma IL-27, **(B)** mRNA expression of the IL-27 signalling pathway in inguinal adipocytes, and **(C)** mRNA expression of the IL-27 signalling pathway in perirenal adipocytes. Data are presented as mean ± SEM. Significant differences between groups are indicated as *p < 0.05, **p < 0.01, and ***p < 0.001.

To investigate the effects of obesity and long-term exercise training on IL-27 expression, we measured plasma IL-27 levels and the mRNA expression of IL-27 and related pathway components in adipose tissue. Plasma IL-27 levels were significantly reduced in obese rats (Figure 5A). Similarly, mRNA expression of IL-27 and associated signalling molecules was markedly reduced in iWAT and PRAT (Figures 5B,C). Exercise training reversed these effects, increasing plasma IL-27 levels and upregulating IL-27 and its regulatory pathway in adipose tissue. Compared with MAIT, HIIT was more effective in elevating plasma IL-27 levels and promoting mRNA expression of IL-27, p38 MAPK, and PGC-1α in adipose tissue. Mechanistically, IL-27 facilitated p38 MAPK phosphorylation, which subsequently enhanced PGC-1α activity. PGC-1α, a key regulator of UCP1-mediated thermogenesis, was thus identified as a critical downstream effector of this pathway (Ricquier and Bouillaud, 2000; Stine et al., 2013; Norheim, 2014).

## 4 Discussion

Exercise training significantly reduced body weight and improved lipid metabolism in obese rats. In both subcutaneous and perirenal adipose tissues, exercise decreased adipose tissue weight and adipocyte size, increased COX and SDH activity, and upregulated BAT-related genes (*Cidea*, *Cited*, *Tbx1*, and *Ucp1*). These findings suggest that exercise enhances mitochondrial enzyme activity and fatty acid oxidation capacity by activating the IL-27/AMPK/PGC-1α pathway, thereby promoting adipose browning and improving metabolic homeostasis.

Exercise training induces a series of endocrine adaptations, including altered secretion of pro- and anti-inflammatory cytokines. Its effects are bidirectional: Moderate exercise exerts anti-inflammatory effects, whereas excessive exercise may elevate inflammatory responses. Long-term exercise training activates the systemic immune system, increasing circulating leukocytes, activating innate and adaptive immune cells in peripheral blood, and upregulating cytokines such as IL-6, IL-10, IL-8, and IL-1RA (Pedersen and Toft, 2000), thereby balancing inflammation and improving insulin sensitivity. IL-27 shares similar properties. As a heterodimer composed of Epstein–Barr virus-induced 3 (EBI3) and p28, IL-27 modulates immune responses through pleiotropic effects (Yoshida and Hunter, 2015). Under stress and basal conditions, IL-27 plays dual roles: Its pro-inflammatory role is mediated via STAT1 and STAT4 activation, promoting Th1 differentiation and IFN-γ production, while its anti-inflammatory role involves STAT3 and STAT5 activation, which suppresses T-cell activation and inflammatory cytokine secretion (Villarino et al., 2004). Obesity reduces endogenous IL-27 levels, consistent with our findings, whereas exercise training reverses this reduction. Restoration of IL-27 levels induced by HIIT activates the p38 MAPK–PGC-1α signalling pathway, with PGC-1α serving as a key regulator of UCP1 expression and thermogenesis (Ricquier and Bouillaud, 2000; Stine et al., 2013; Norheim, 2014). UCP1, a critical marker of adipose browning, is thus directly upregulated via IL-27-driven PGC-1α activity. Additionally, PGC-1α promotes mitochondrial biogenesi (Trevellin et al., 2014). In transgenic mouse models, skeletal muscle-specific overexpression of PGC-1α significantly increased UCP1 mRNA and protein levels in subcutaneous adipose tissue, and electron microscopy revealed a marked increase in mitochondrial number, directly demonstrating PGC-1α′s role in promoting mitochondrial biogenesis (Boström et al., 2012). Together, increased mitochondrial abundance and activity are key features of adipose browning.

In this study, the HIIT and MAIT exercise protocols were designed based on accurate assessment of rats’ maximal aerobic capacity, aiming to simulate different intensity exercise patterns in humans. Compared with MAIT, HIIT—characterised by alternating high- and low-intensity phases—more effectively modulated systemic inflammation and activated the p38 MAPK–PGC-1α pathway, thereby amplifying IL-27-mediated adipose browning. Consequently, UCP1 expression in subcutaneous and perirenal adipose tissues was higher following HIIT than MAIT. Owing to the superior metabolic efficiency of HIIT in upregulating UCP1 expression, HIIT may be recommended as a preferred fat-reduction strategy for individuals with obesity, particularly those with visceral adiposity.

This study has two key limitations. First, it used only male rats and did not account for the potential effects of estrogen on fat metabolism, exercise response, and immune pathways, meaning the findings cannot be directly extrapolated to female populations. Second, the study collected samples immediately after the training intervention, lacking long-term follow-up data. This prevents an assessment of the durability of exercise benefits or the presence of a “metabolic memory” effect, thereby limiting its value for guiding chronic obesity management strategies. Future studies should elucidate the cellular sources of IL-27, its dynamic regulation during exercise, and its post-training level dynamics to determine the duration of HIIT’s effects.

## 5 Conclusion

Exercise training increases IL-27 levels, activates the p38 MAPK–PGC-1α signalling pathway, and promotes adipocyte browning and thermogenesis. These adaptive changes are dependent on exercise intensity.

Compared with MAIT, HIIT more effectively induces WAT browning and thermogenesis, highlighting its potential as a non-pharmacological therapeutic strategy for metabolic disease management.

## Data Availability

The datasets [GENERATED/ANALYZED] for this study can be found in the [Jianguoyun] [https://www.jianguoyun.com/p/DQAJNJIQo475DRjOoJUGIAA].

## References

[B1] Alford S. Patel D. Perakakis N. Mantzoros C. S. (2018). Obesity as a risk factor for Alzheimer’s disease: weighing the evidence. Obes. Rev. 19 (2), 269–280. 10.1111/obr.12629 29024348

[B2] Boström P. Wu J. Jedrychowski M. P. Korde A. Ye L. Lo J. C. (2012). A PGC1-α-dependent myokine that drives brown-fat-like development of white fat and thermogenesis. Nature 481 (7382), nr463–nr468. 10.1038/nature10777 22237023 PMC3522098

[B3] Chang B. Song C. Gao H. Ma T. Li T. Ma Q. (2021). Leptin and inflammatory factors play a synergistic role in the regulation of reproduction in male mice through hypothalamic kisspeptin-mediated energy balance. Reproductive Biol. Endocrinol. 19 (1), nr12. 10.1186/s12958-021-00698-0 33472656 PMC7816398

[B4] Chen X. Gui G. Ji W. Xue Q. Wang C. Li H. (2019). The relationship between obesity subtypes based on BMI and cardio-cerebrovascular disease. Hypertens. Res. 42 (6), 912–919. 10.1038/s41440-018-0184-4 30622319

[B5] Edgar D. Shabalina I. Camara Y. Wredenberg A. Calvaruso M. A. Nijtmans L. (2009). Random point mutations with major effects on protein-coding genes are the driving force behind premature aging in mtDNA mutator mice. Cell. Metab. 10 (2), nr131–nr138. 10.1016/j.cmet.2009.06.010 19656491

[B6] Høydal M. A. Wisløff U. Kemi O. J. Ellingsen O. (2007). Running speed and maximal oxygen uptake in rats and mice: practical implications for exercise training. Eur. J. Cardiovasc. Prev. and Rehabilitation 14 (6), 753–760. 18043295 10.1097/HJR.0b013e3281eacef1

[B7] Khalafi M. Mohebbi H. Symonds M. E. Karimi P. Akbari A. Tabari E. (2020). The impact of moderate-intensity continuous or high-intensity interval training on adipogenesis and browning of subcutaneous adipose tissue in obese Male rats. Nutrients 12 (4), nr925. 10.3390/nu12040925 32230849 PMC7231004

[B8] Kim N. H. Jung Y. S. Park J. H. Park D. I. Sohn C. I. (2019). Abdominal obesity is more predictive of advanced colorectal neoplasia risk than overall obesity in men: a cross-sectional study. J. Clin. Gastroenterology. 53 (7), e284–e290. 10.1097/MCG.0000000000001086 29939870

[B9] Lee M. O. (1929). Determination of the surface area of the white rat with its application to the expression of metabolic results. Am. J. Physiology-Legacy Content 89 (1), nr24–nr33. 10.1152/ajplegacy.1929.89.1.24

[B10] Leggio M. Lombardi M. Caldarone E. Severi P. D”Emidio S. Armeni M. (2017). The relationship between obesity and hypertension: an updated comprehensive overview on vicious twins. Hypertens. Res. 40 (12), 947–963. 10.1038/hr.2017.75 28978986

[B11] Liu P. H. Wu K. Ng K. Zauber A. G. Nguyen L. H. Song M. (2019). Association of obesity with risk of early-onset colorectal cancer among women. Am. Med. Assoc. 5 (1), 37–44. 10.1001/jamaoncol.2018.4280 30326010 PMC6382547

[B12] Nam H. Ferguson B. S. Stephens J. M. Morrison R. F. (2016). Modulation of IL ‐27 in adipocytes during inflammatory stress. Obesity 24 (1), nr157–nr166. 10.1002/oby.21351 26638127 PMC4688214

[B13] Norheim F. Langleite T. M. Hjorth M. Holen T. Kielland A. Stadheim H. K. (2014). The effects of acute and chronic exercise on PGC‐1α, irisin and browning of subcutaneous adipose tissue in humans. FEBS J. 281 (3), 739–749. 10.1111/febs.12619 24237962

[B14] Pedersen B. K. Toft A. D. (2000). Effects of exercise on lymphocytes and cytokines. Br. J. Sports Med. 34 (4), nr246–nr251. 10.1136/bjsm.34.4.246 10953894 PMC1724218

[B15] Ricquier D. Bouillaud F. (2000). The uncoupling protein homologues: UCP1, UCP2, UCP3, StUCP and AtUCP. Biochem. J. 345 (2), nr161–nr161. 10620491 PMC1220743

[B16] Ross J. M. (2011). Visualization of mitochondrial respiratory function using cytochrome c oxidase/succinate dehydrogenase (COX/SDH) double-labeling histochemistry. J. Vis. Exp. 57, e3266. 10.3791/3266 22143245 PMC3308593

[B17] Ross J. M. Oeberg J. Brene S. Coppotelli G. Terzioglu M. Pernold K. (2010). High brain lactate is a hallmark of aging and caused by a shift in the lactate dehydrogenase A/B ratio. Proc. Natl. Acad. Sci. 107 (46), nr20087–nr20092. 10.1073/pnas.1008189107 PMC2993405 21041631

[B18] Sidossis L. Kajimura S. (2015). Brown and beige fat in humans: thermogenic adipocytes that control energy and glucose homeostasis. J. Clin. Investigation 125 (2), nr478–nr486. 10.1172/JCI78362 25642708 PMC4319444

[B19] Stine R. Jakob G. K. Lotte L. Anders L. Maja M. N. Henriette P. (2013). PGC-1α is required for Exercise- and exercise training-induced UCP1 Up-Regulation in mouse white adipose tissue. Plos One 8 (5), nre64123. 10.1371/journal.pone.0064123 PMC3661446 23717545

[B20] Trevellin E. Scorzeto M. Olivieri M. Granzotto M. Valerio A. Tedesco L. (2014). Exercise training induces mitochondrial biogenesis and glucose uptake in subcutaneous adipose tissue through eNOS-dependent mechanisms. Diabetes 63 (8), nr2800–nr2811. 10.2337/db13-1234 24622799

[B21] Villarino A. V. Huang E. Hunter C. A. (2004). Understanding the Pro- and anti-inflammatory properties of IL-27. J. Immunol. 173 (2), nr715–nr720. 10.4049/jimmunol.173.2.715 15240655

[B22] Wang Q. Li D. Cao G. Shi Q. Zhu J. Zhang M. (2021). IL-27 signalling promotes adipocyte thermogenesis and energy expenditure. Nature 600 (7888), nr314–nr318. 10.1038/s41586-021-04127-5 34819664

[B23] Yi X. Tang D. Cao S. Li T. Gao H. Ma T. (2020). Effect of different exercise loads on testicular oxidative stress and reproductive function in obese Male mice. Oxidative Med. Cell. Longev. 2020, nr1–13. 10.1155/2020/3071658 32082477 PMC7007943

[B24] Yiliang Z. Shengyang Z. Runming Z. Yingzhen H. Yan W. (2024). Chronic cold exposure reprograms feeding-regulated LPL activity in white adipose tissues through hepatic ANGPTL3 and ANGPTL8. Life Metab. (1), nr1. 10.1093/lifemeta/loae037 PMC11770819 39872988

[B25] Yoshida H. Hunter C. A. (2015). The immunobiology of Interleukin-27. Annu. Rev. Immunol. 33 (1), nr417–nr443. 10.1146/annurev-immunol-032414-112134 25861977

[B26] Yu J. Zhang S. Cui L. Wang W. Na H. Zhu X. (2015). Lipid droplet remodeling and interaction with mitochondria in mouse brown adipose tissue during cold treatment. Biochimica Biophysica Acta (BBA) - Mol. Cell. Res. 1853 (5), nr918–nr928. 10.1016/j.bbamcr.2015.01.020 25655664

